# Supplementary figures and images for: The multifaceted role of EXOC6A in ciliogenesis
Source: eLife. 2026 May 20;14:RP108271. doi: 10.7554/eLife.108271 (PMC13189621; doi:10.7554/eLife.108271)

Source Data

Fig.4A

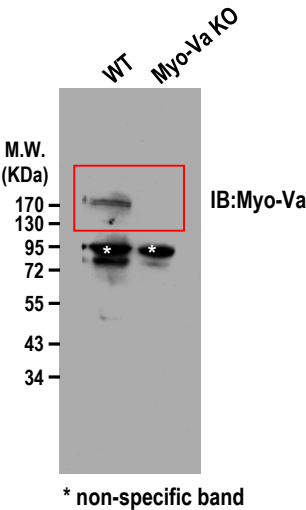

Fig. 4B

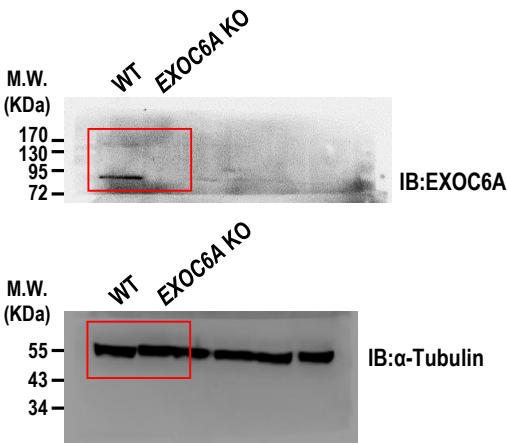

Fig. 4C

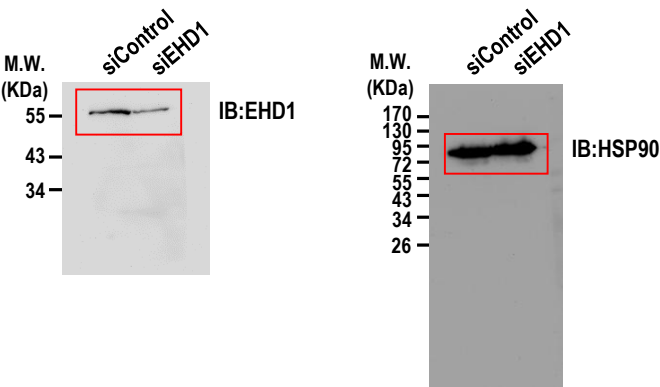

Fig. 4E

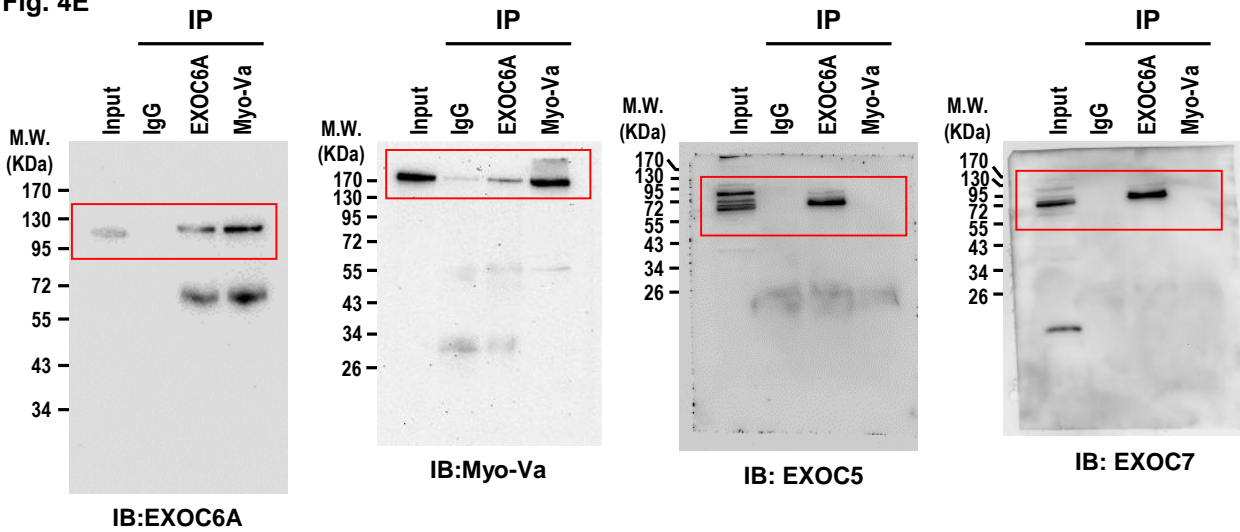

Supplement: Figure 4—source data 1. [file elife-108271-fig4-data1.zip › Figure 4-source data 1.pdf]

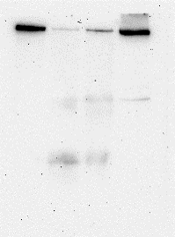

Supplement: Figure 4—source data 2. [file elife-108271-fig4-data2.zip › Figure 4-source data 2-4E-IBVa.tif]

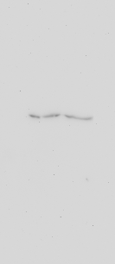

Supplement: Figure 4—source data 2. [file elife-108271-fig4-data2.zip › Figure 4-source data 2-4A-IBhsp90.tif]

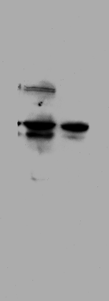

Supplement: Figure 4—source data 2. [file elife-108271-fig4-data2.zip › Figure 4-source data 2-4A-IBMyoVa.tif]

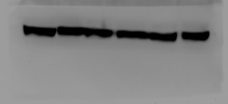

Supplement: Figure 4—source data 2. [file elife-108271-fig4-data2.zip › Figure 4-source data 2-4B-IBatub.tif]

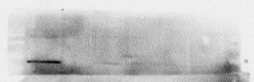

Supplement: Figure 4—source data 2. [file elife-108271-fig4-data2.zip › Figure 4-source data 2-4B-IBEXOC6A.tif]

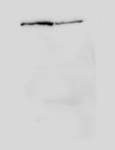

Supplement: Figure 4—source data 2. [file elife-108271-fig4-data2.zip › Figure 4-source data 2-4C-IBEHD1.tif]

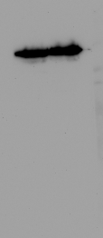

Supplement: Figure 4—source data 2. [file elife-108271-fig4-data2.zip › Figure 4-source data 2-4C-IBhsp90.tif]

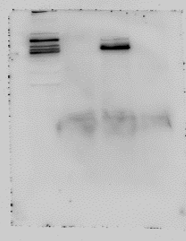

Supplement: Figure 4—source data 2. [file elife-108271-fig4-data2.zip › Figure 4-source data 2-4E-IBEXOC5.tif]

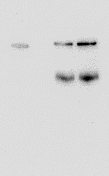

Supplement: Figure 4—source data 2. [file elife-108271-fig4-data2.zip › Figure 4-source data 2-4E-IBEXOC6.tif]

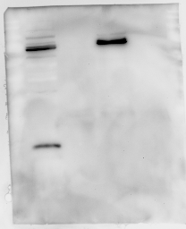

Supplement: Figure 4—source data 2. [file elife-108271-fig4-data2.zip › Figure 4-source data 2-4E-IBEXOC7.tif]

Figure 4—figure supplement 1C

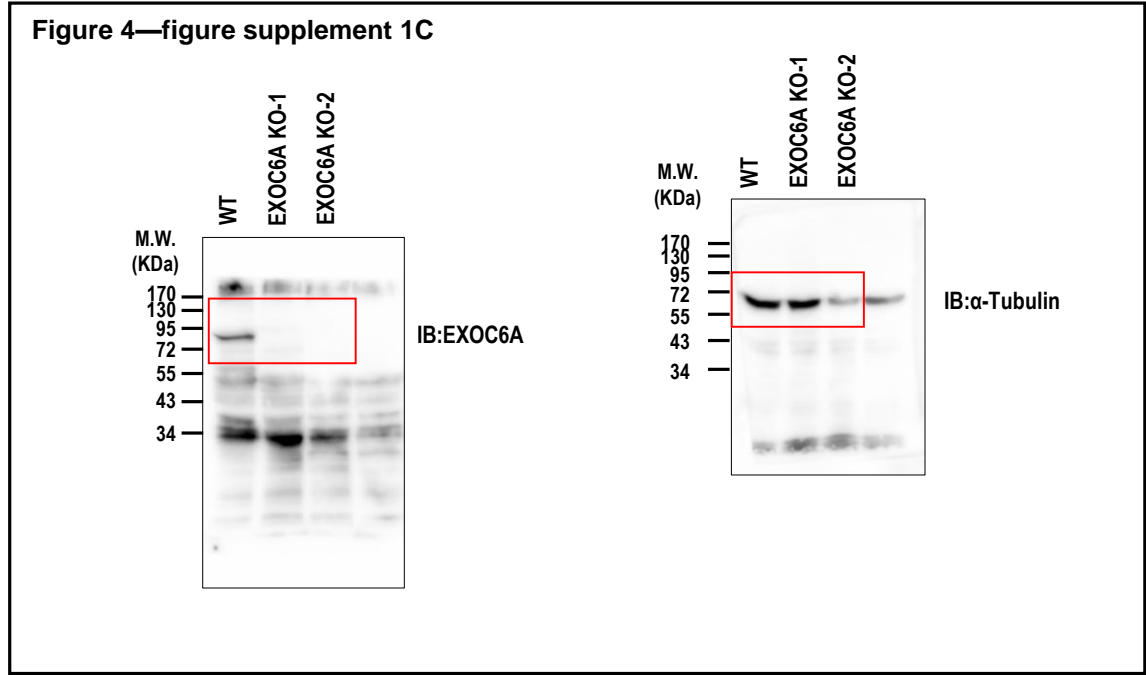

Supplement: Figure 4—figure supplement 1—source data 1. [file elife-108271-fig4-figsupp1-data1.zip › Figure 4-figure supplement 1-source data 1.pdf]

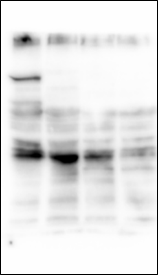

Supplement: Figure 4—figure supplement 1—source data 2. [file elife-108271-fig4-figsupp1-data2.zip › Figure 4-figure supplement 1-source data 2-1C-IB6A.tif]

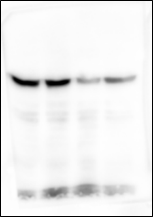

Supplement: Figure 4—figure supplement 1—source data 2. [file elife-108271-fig4-figsupp1-data2.zip › Figure 4-figure supplement 1-source data 2-1C-IBatub.tif]

Source Data

Fig. 5B

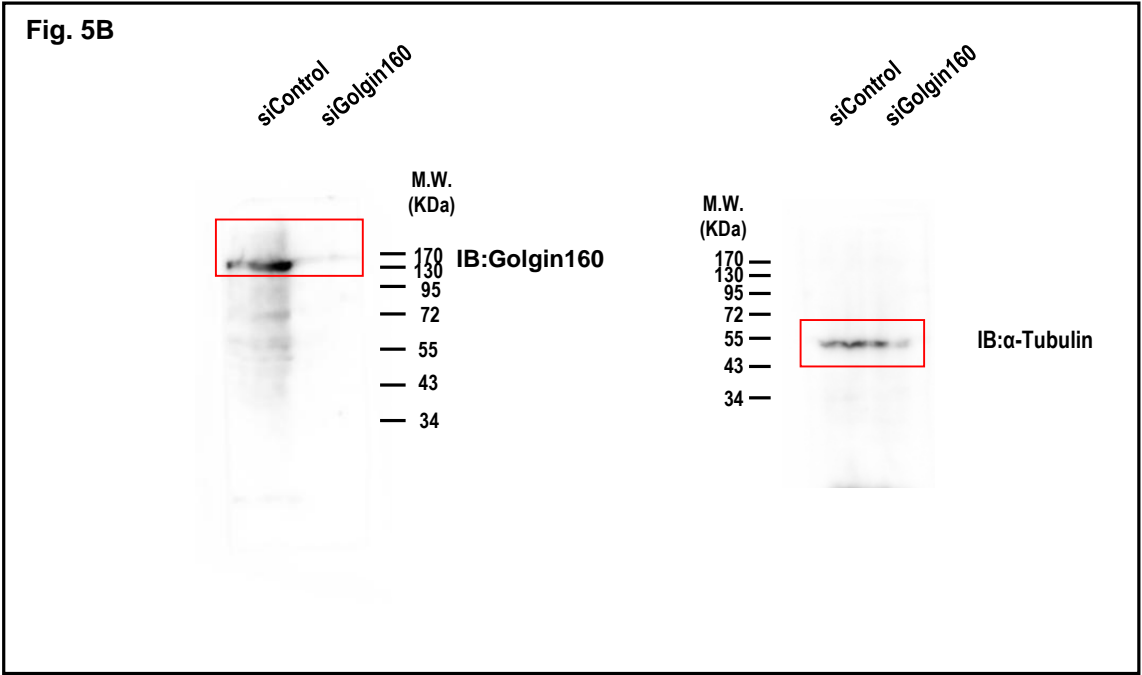

Fig. 5G

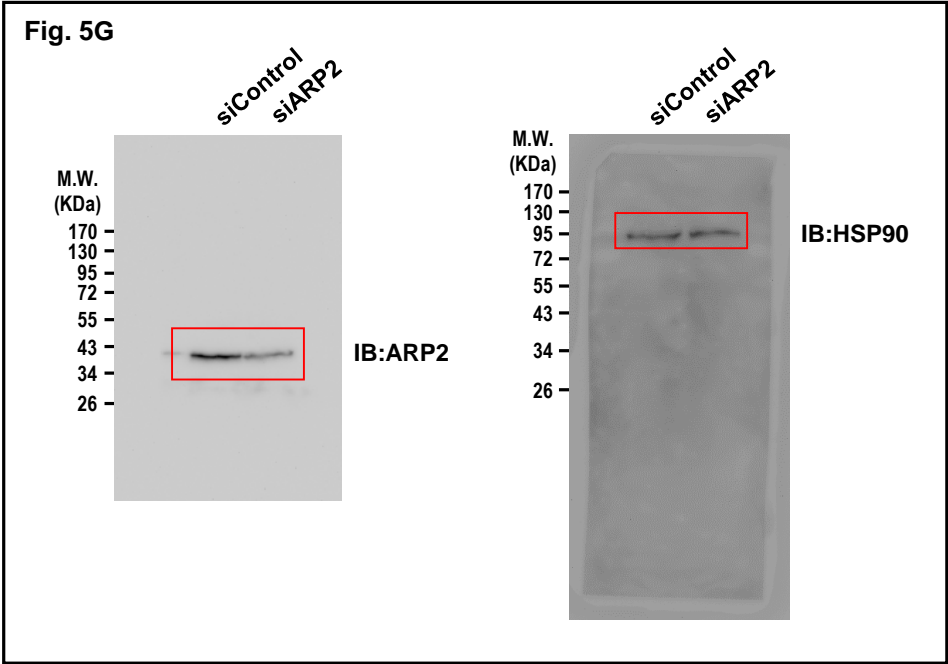

Supplement: Figure 5—source data 1. [file elife-108271-fig5-data1.zip › Figure 5-source data 1.pdf]

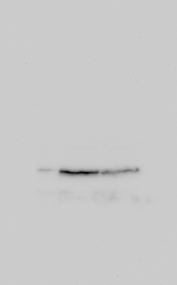

Supplement: Figure 5—source data 2. [file elife-108271-fig5-data2.zip › Figure 5-source data 2-5G-IBarp2.tif]

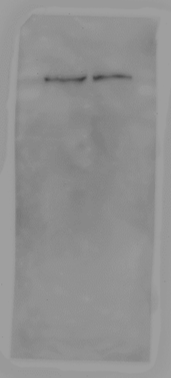

Supplement: Figure 5—source data 2. [file elife-108271-fig5-data2.zip › Figure 5-source data 2-5G-IBhsp90.tif]

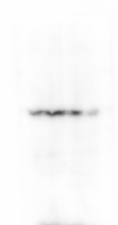

Supplement: Figure 5—source data 2. [file elife-108271-fig5-data2.zip › Figure 5-source data 2-5B-IBaTub.tif]

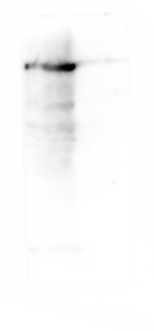

Supplement: Figure 5—source data 2. [file elife-108271-fig5-data2.zip › Figure 5-source data 2-5B-IBGolgin160.tif]
